# [Retracted] Resveratrol suppresses melanoma growth by promoting autophagy through inhibiting the PI3K/AKT/mTOR sigaling pathway

**DOI:** 10.3892/etm.2024.12668

**Published:** 2024-07-30

**Authors:** Changhua Gong, Honglei Xia

Exp Ther Med 19:1878–1886, 2020; DOI: 10.3892/etm.2019.8359

Following the publication of this paper, it was drawn to the Editors’ attention by a concerned reader that the cell migration and invasion assay data shown in Fig. 2A, C, E and G on p. 1182, the immunofluorescence data shown in Fig. 3A on p. 1183, and the Transwell cell migration assay data shown in Fig. 6D on p. 1185 were strikingly similar to data appearing in different form in other articles written by different authors at different research institutes that had already been published elsewhere prior to the submission of this paper to *Experimental and Therapeutic Medicine*, or which were under consideration for publication at around the same time (several of which have been retracted). In view of the fact that the abovementioned data had already apparently been published previously, the Editor of *Experimental and Therapeutic Medicine* has decided that this paper should be retracted from the Journal. The authors were asked for an explanation to account for these concerns, but the Editorial Office did not receive a reply. The Editor apologizes to the readership for any inconvenience caused.

